# A preliminary study: designing and validating projective images of Young’s early maladaptive schema (EMS) domains

**DOI:** 10.1186/s40359-021-00514-9

**Published:** 2021-01-28

**Authors:** Javad Siahmoshtei, Ali Delavar, Ahmad Borjali

**Affiliations:** Allameh Tabataba’i University, Tehran, Iran

**Keywords:** Validation, Projective image, EMS domains

## Abstract

**Background:**

This study aims to design and validate ten projective images of *Young’s Early Maladaptive Schema *(*EMS*) domains. For this purpose, two questions are to be addressed. (1) How is the factorial structure of the projective images of EMS domains? (2) Do the images designed in the domains of disconnection and rejection, impaired autonomy and performance, impaired limits, other-directedness, and over-vigilance and inhibition have sufficient validity?

**Methods:**

This is an applied mixed-methods exploratory study, in which the statistical population consisted of psychologists from Tehran Province in the qualitative section (n = 8) as well as other individuals aged between 18 and 65 years (mean age = 33) from Qazvin in the quantitative section (n = 102) in 2018. The research questions were analyzed through principal axis factoring with a varimax rotation, confirmatory factor analysis, Pearson correlation coefficient, and Cronbach’s alpha.

**Results:**

According to the results, ten images and five domains of Young’s EMSs contribute to a simple structure. Accounting for 70.35% of the total variance of EMSs, the five dimensions include disconnection and rejection, impaired autonomy and performance, impaired limits, other-directedness, and over-vigilance and inhibition.

**Conclusions:**

The results indicated that the designed projective images yielded acceptable construct validity.

## Background

Psychology is recognized as an independent and empirical science when it tests, operationally defines, and measures psychological phenomena [51]. Tests are the most common tools for measuring psychological characteristics and traits [46]. Psychologists conduct personality tests to identify clients’ behavior, thoughts, and emotions. In general, personality tests are divided into self-report clinical questionnaires and projective tests. Self-report questionnaires containing standardized items with specific responses were initially used to measure psychological traits and characteristics [26]. These questionnaires are also called objective tests, for the rater’s subjective judgment is not involved in the rating process [66]. Allard and Faust (2000) claimed that a source of threat for objective tests would be the scoring error and that there would not be such a large number of psychometric papers on different styles of responses and biases of these types of tests if they were really objective. More than half a century ago, Meehl (1945) argued that such factors as inherent ambiguity of test items, constraints on self-knowledge or self-perception, personal dynamics, and even projections would affect respondents’ answers to self-report questionnaires [34]. In the late 1930s and early 1940s, organized personality tests were gradually replaced by projective tests prepared with vague stimuli and unspecified answers [26]. Projective tests are based on the idea that people are unable or unwilling to provide accurate information in objective or self-report questionnaires [66]. Based on the projection hypothesis, the projective test was defined by Frank as “when people try to understand vague stimuli, their interpretation of these stimuli can reflect their feelings, needs, experiences, previous conditioned behavior, and thinking processes.” [51]. These tests regard the personality as a whole unit that is undividable into elements [21]. Therefore, the items of a projective test act as a cinema screen on which the respondents project their anxiety, conflicts, and psychological needs [3]. The thematic apperception test (TAT) is a type of projection technique with 30 images to measure 28 needs based on Murray’s theory. The respondents’ answers in the narratives that they make up about ambiguous pictures reveal their underlying thoughts and feelings. This technique is also used for children in the children's apperception test (CAT) and for the elderly in the senior appreciation test (SAT) by changing image types on cards. Specific to minorities, the “Tell Me a Story” test contains 23 cards to extract interpersonal conflicts, whereas the Rosenzweig picture frustration test has been designed to show respondents’ frustration and how they experience and cope with it [24]. Serfass and Sherman [50] analyzed the TAT situation perception, the results of which supported a two-component view including an objective component that could be attributed to perception conditions and a subjective component that could be attributed to the subject. Okamoto et al. [36] employed the projective PPM images to measure stereotypes and described the results as acceptable and objective. Leite et al. [31] assessed the pictorial test of cognitive profiles (TPPC) and proved the results reliable.

Cognitive scientists agree that mental knowledge consists of mental representation [62] including mental imagery of objects, events, and situations experienced in the past. The mental representation can also include such senses as sight, hearing, taste, and touch. The bulk of studies conducted on cognitive psychology of mental representation have focused on visual representations [62]. A noticeable finding was obtained from a study conducted by Martha Farah, a professor at the University of Pennsylvania. Her study showed that many brain parts involved in visual perception were also involved in mental representation [57]. Minsky proposed the concept-like frames of representation for the first time ever in the 1970s. These frames were called schemas and scripts by researchers in the fields of artificial intelligence and psychology [62]. In fact, they refer to the existence of a general organization and board that control, organize, and give meaning to individuals’ mental representation and perception from their surroundings. They are also considered among the deep cognitive and personality-related layers of individuals.

The word “schema” comes from a Greek word referring to an organized pattern or structure contributing to order in a complex set of stimuli and experiences. In psychology, the term schema was used for the first time ever in the area of cognitive development and was then extended to cognitive therapy. In cognitive psychology, a schema is a mental cognitive map utilized to interpret information and solve a problem [39].

Including a range of meaningfully relevant organized concepts, schemas are mental frameworks for representing knowledge [57]. According to Rumelhart and Ortony (1977) and Thorndike (1984), schemas have specific features which make them flexible. (A) Schemas can include other schemas. (B) Inclusive schemas are typical and general realities. (C) Schemas vary in abstraction [57]. Evidently, schemas are an integral part of cognitive, emotional, and social actions of human beings, whereas individuals come to recognize the world around them through schemas as if they come into being in their schematic worlds.

Accordingly, EMSs are the comprehensive patterns of memories, emotions, cognition, and physical emotions that include self-understanding and relationships with others [47]. Repeated over the course of life, EMSs are self-harming emotional and cognitive patterns formed in the mind from the beginning of growth and development. Accordingly, a person's behavior is not considered a part of a schema, for Young believes that maladaptive behavior emerges in response to schemas. Therefore, behavior emerges from schemas, however, they are not part of them [67].

The evolutionary roots of EMSs lie in the unpleasant childhood experiences. Early-stage schemas are usually stronger [40]. Formed from the early ages, EMSs are stable and severely disruptive throughout life [47]. Many studies have considered EMSs a major factor disturbing and threatening mental health [47].

Some studies have shown that EMSs are involved in vulnerabilities to mental disorders and problems [13].

According to other studies, the schema therapy approach is efficient in treating chronic anxiety, eating disorders, depression, and marital problems as well as in maintaining intimate relationships and preventing relapse in drug users and criminals [22].

The results of recent studies also show that the use of schema therapy can improve depression [8, 43] and hyperactivity in patients with autism [38] and help deal with stress and responsibility in the caregivers of schizophrenic patients [54], dysfunctional attitudes and also enhance coping strategies in patients with body dysmorphic disorder [14] and mental health [60]. Cognitive therapists believe that a schema or, in general, cognition plays a key role in the formation of human behavior. In other words, human actions depend on their rate of thoughts and emphasis on misconceptions regarding the source of psychological problems.

Schema therapy consists of two main stages, the first of which includes measurement and education, whereas the second is intervention. The measurement stage consists of the most basic activity provided by the schema therapist to treat and alleviate the patients’ maladaptive schemas and continue the treatment process. The schema questionnaire is a tool used in this therapeutic approach, whereas and the *Schema Questionnaire-Short Form* (*SQ-SF*) is a very useful instrument for measuring EMSs [37]. Lee et al. [30] analyzed the psychometric properties of this questionnaire in South Korea, Staniaszek and Popiel [56] in Poland, Lyrakos [32] in Greece, Kriston et al. [27] in Germany, Cazassa and Oliveira [10] in Brazil, Rijkeboer and van den Bergh [44] in the Netherlands, and [55] in Romania, All of them reported the psychometric properties of the SQ-SF to be appropriate. Kriston et al. [28] reported no clear evidence confirming the schema domains. Bach et al. [5] reported four schema domains in the Danish community in an empirical and conceptual sense based on Young’s schema therapy model, personality pathology, and chronic emotional disorders. Aloi et al. [2] analyzed the YSQ-S3 scale in an Italian society and reported it a reliable instrument with appropriate construct validity. Thus, they confirmed the existence of four schema domains based on the evidence. Several studies have reported satisfactory results for the psychometric properties of the second version of the SQ-SF in Iran between 2005 and 2009. However, its third version was only analyzed by Ghiasi [22] in Iran who reported findings in line with Young’s schema theory. Nevertheless, the important point about all these studies on the psychometric properties of the second and third short form versions of this questionnaire in different societies would be the varying number of extracted schemas. However, the present study aims to identify schemas through their respective domains in the visual projective method in which the images are designed based on the main theme of schema domains.

The EMS questionnaire is also very time-consuming both in its implementation and in its clinical examination by clinicians; however, respondents may not cooperate properly in completing the questionnaire. Therefore, aiming at the visual projective design of maladaptive schemas in 10 cards, this study intends to accelerate the process of measuring schemas and elicit greater willingness and participation from the clients.

A question may now arise. Can the projective images, with regard to the theory behind them and the findings of cognitive science, be used as appropriate tools for measuring this part of individuals’ deep psychological problems?

From the perspective of schema therapy, the measurement and evaluation of EMSs are important activities. Young identified 18 schemas in five domains including disconnection and rejection, impaired autonomy and performance, impaired limits, other-directedness, and over-vigilance and inhibition. Failure to meet the basic emotional needs would be an important factor in the schemas related to each domain. Bach et al. [5] concluded that the four higher-order domains of “disconnection and rejection”, “impaired autonomy and performance”, “excessive responsibility and standards”, and “impaired limits” are differentially associated with conceptually relevant need-thwarting parental experiences.

Therefore, this study seeks to design projective images for measuring these five main domains. Other questions also arise. Is it possible to design projective images which can activate the existing schemas of individuals? Can these images be used valid tools to reflect individuals’ schemas in the form of a story in response to images? Is it possible to employ such tools to measure the schema domains instead of measuring each schema separately (by means of a questionnaire), thereby saving time and cost? And will the individuals’ schemas reveal themselves in the face of these images as they do in response to different stimuli in different life and work situations regarded as a fixed mental phenomenon and a cognitive framework? Hence, this study aims to design and validate ten projective images related to Young’s EMS domains by designing two cards A and B for each domain. “A” cards are based on individuals’ basic emotional needs and early life experiences, whereas “B” cards are based on their basic emotional needs and everyday life situations in adulthood. The study also intends to test and answer the above questions. The final and main research question is whether it is possible to measure the subjects’ EMS domains by analyzing the stories associated with the “A” and “B” images of each domain.

## Methods

This is an applied (developmental) mixed-methods exploratory study, in which a conceptual model was first depicted by using the qualitative method, interviews, and coding. Data were then collected and analyzed through the quantitative method including principal axis factoring (PAF) with a varimax rotation, confirmatory factor analysis (CFA), PPMCC, and Cronbach’s alpha. The statistical population consisted of two groups, the first of which included psychology experts from Tehran Province, whereas the second group included adults from Qazvin Province to verify the construct validity of the projective images. The research sample groups of experts consisted of 11 experts [48] including three experts in psychometrics, four clinical psychologists, and four experts in counseling psychology. The research sample group of adults consisted of 102 people including 52 males and 50 females aged between 18 and 65 years. The purposive sampling method was used for in the expert group, whereas the convenience sampling method was employed in the adult group. The data collection tool included ten projective cards and a standard questionnaire. To determine Young’s EMS domains, the researcher used ten projective images measuring the five domains proposed by Young. Two cards were considered in each domain including an A card based on one’s basic emotional needs and early life experiences as well as a B card based on one’s basic emotional needs and everyday situations in adult life. The researcher also used the *Young Schema Questionnaire (YSQ-S3)* (2005), i.e. a 90-item self-report tool for measuring 18 schemas in five domains. The items were scored on a 6-point Likert scale (from 1: completely wrong to 6: completely right) [22]. The factor analysis showed that the questionnaire had good construct validity and that the main five domains (disconnection and rejection, impaired autonomy and performance, impaired limits, other-directedness, over-vigilance and inhibition) were extracted. Cronbach’s alpha of the questionnaire was reported 0.94. The researcher also used the exploratory factor analysis (EFA) through PAF with a varimax rotation. The premises of sufficient sample size and multiple linearity were analyzed through the KMO statistic and the Bartlett’s test of sphericity. The divergent validity was also measured through the average variance extracted (AVE) index, reliability with Cronbach’s alpha, and combined reliability (CR), whereas and the convergent validity was checked through the Fornell–Larcker matrix and PPMCCs in SPSS 23. The structural equation modeling was then employed along with the partial least squares approach in SmartPLS 2 for the confirmatory factor analysis.

### Measures

The researcher had a designer design the projective images of concepts in Young’s EMS domains, for which the necessary training was received from the projection techniques. The thematic apperception test (TAT) was also used as an exemplar of this type of measurement technique. Having analyzed the design and style of projection imagery, the designer was acquainted with the research concepts including the domains and schemas as well as the necessary design. The researcher also set forth ideas regarding each domain in order to facilitate and expedite the design and imagery in each domain to be mixed with the designer’s imagery. In the next step, after the images were designed in each domain, they were presented to the researcher’s supervisor, who was an expert in psychology and projection techniques. The supervisor removed the problems with the theory of the projection technique as well as the content of the schema domains in the designed images. Finally, ten relatively suitable projection images were obtained—each was related to a specific domain—in several face-to-face meetings and through virtual training over a period of several months. In the next step, after approval by the supervisor, several other experts also examined the designs in terms of content and face validity. For this purpose, the designs were presented along with a survey form to six clinical psychologists and faculty members from Tehran. In total, professors and experts who criticized and evaluated the content and face validity of the images were as follows: three experts in psychometrics, four clinical psychologists, and one counseling specialist. The designed images were then executed on 102 people among the YSQ-S3 respondents from the statistical population in the clinic with psychiatric and counseling services during a three-month period. For performing and scoring cards, TAT instructions were used [24] with differences of stories presented by the respondents based on the emotional needs of Jeffrey Young’s theory. The primary maladaptive schemas were also assessed and recognized.

## Results

### Demographic characteristics

The following results were obtained from 102 respondents in this study:

There were 50 female respondents (49%) and 52 male ones (51%). Moreover, seven respondents (6.9%) were below 20 years old, whereas 36 of them (35.3%) were aged between 21 and 30 years old. Furthermore, 41 respondents (40.2%) were aged between 31 and 50 years old, and 16 of them (15.7%) were aged between 41 and 50 years old. Only two respondents (2%) were above 50 years old. Two respondents (2%) finished the third grade of middle school, whereas 21 of them (20.6%) had (high school) diplomas, and 63 respondents (61.8%) had bachelor’s degrees. In addition, 16 respondents (15.7%) had master’s degrees. In terms of language, 69 respondents (67.6%) spoke Persian, whereas 26 (25.5%) spoke Turkish, and 7 (6.9%) spoke other languages.

Question 1 – How is the factorial structure of the projective images of the EMS domains?

The EFA-based approach was used along with PFA and a varimax rotation in SPSS 23 to identify and determine the factors underlying the performance in the sub-tests. The Bartlett’s test was conducted to determine the sample size sufficiency, whereas the Kaiser criterion was employed to determine the number of factors. In order to use the factor analysis, the researcher first examined the following assumptions.

### Sample size sufficiency and linear multiplicity assumptions

The Kaiser–Meyer–Olkin statistic was reported 0.69. The acceptable KMO value equal to and greater than 0.60; therefore, the first assumption was proven true. If the KMO index is above 0.60, the sample size is sufficient.

### Similarities of each card to the whole test

According to the results presented in Table 1, the values related to the total extracted similarities of all cards for the factor analysis are above 0.25. This indicates an acceptable correlation between each card and the whole test and its suitability for factor analysis.

**Table 1:** The similarity degree of each card with the whole test

Card	Initial	Extracted
CARD1A	1	0.787
CARD1B	1	0.716
CARD2A	1	0.796
CARD2B	1	0.689
CARD3A	1	0.73
CARD3B	1	0.73
CARD4A	1	0.634
CARD4B	1	0.681
CARD5A	1	0.616
CARD5B	1	0.663

### Determining the number of extracted factors

The best factor extraction method to use Kaiser’s criterion, according to which factors whose eigenvalues are greater than 1 are considered acceptable. As the first column of Table 2 presents, based on the Kaiser’s criterion, the lambda value (eigenvalue) was greater than 1 for five factors. When the lambda value is greater than one, it can be considered a main factor. Therefore, there are only five main factors of these cards with which a simple appropriate structure can be obtained.

**Table 2:** Total sum of variance based on the PAF of the projective images of Young’s EMS domains

Construct	Initial eigenvalues			Total squares of the initial extracted loads			Total squares of the extracted loads after rotation		
	Total	Variance ratio	Cumulative variance ratio	Total	Variance ratio	Cumulative variance ratio	Total	Variance ratio	Cumulative variance ratio
1	1.823	18.225	18.225	1.823	18.225	18.225	1.570	15.699	15.699
2	1.524	15.240	33.465	1.524	15.240	33.465	1.548	15.484	31.183
3	1.319	13.192	46.658	1.319	13.192	46.658	1.355	13.548	44.731
4	1.279	12.791	59.449	1.279	12.791	59.449	1.332	13.316	58.046
5	1.090	10.903	70.351	1.090	10.903	70.351	1.231	12.305	70.351
6	0.786	7.864	78.216						
7	0.694	6.936	85.152						
8	0.582	5.819	90.971						
9	0.511	5.108	96.078						
10	0.392	3.922	100.000						

As discussed earlier, the correlation matrix was used along with PAF and a varimax rotation. Table 2 indicates that the five listed factors account for 70.35% of the total variance of the EMS domains after the rotation (the first factor 15.70%, the second factor 15.48%, the third factor 13.55%, the fourth factor 13.32%, and the fifth factor 12.30%). Accordingly, considering the five factors for these images, it is possible to develop a simple appropriate structure with only 29.65% of the data being lost.

The factor matrix of the data reached the best structure of images after 250 times of test rotation. Table 3 shows the results, according to which each image has a factor load of above 40% at least on one of the factors. There is also no image that has the same or a close factor load on two or more factors simultaneously. This indicates the appropriateness and acceptability of the extracted factors and factor loads of images. Therefore, by considering five factors for 10 images, a simple and suitable structure is achieved for these images. This structure is consistent with the theoretical foundations and content validity of images.

**Table 3:** Matrix of the extracted constructs (factors) of the projective images of Young’s EMS domains after rotation

Image	Factor				
	1	2	3	4	5
CARD3A	0.825				
CARD3B	0.743				
CARD1A		0.862			
CARD1B		0.827			
CARD4B			0.812		
CARD4A			0.754		
CARD5B				0.779	
CARD5A				0.751	
CARD2A					0.876
CARD2B					0.629

### Naming the factors

To examine the simple structure of the projective images of Young’s EMS domains, the extracted factors underwent the varimax rotation. Table 3 shows the factor loads of the four extracted factors, i.e. correlations between factors and images. The minimum significant factor load is considered 40% in this study. Each image had a greater factor load on one of the extracted factors and a smaller factor load on the rest of the factors. According to Table 4, the images related to each of the factors are as follows:

- First factor: Two images including 3A and 3B represent the impaired limits domain.
- Second factor: Two images including 1A and 1B represent the disconnection and rejection domain.
- Third factor: Two images including 4A and 4B represent the other-directedness domain.
- Fourth factor: Two images including 5A and 5B represent the over-vigilance and inhibition domain.
- Fifth factor: Two images including 2A and 2B represent the impaired autonomy and performance domain.

**Table 4:** Extracted factors of the projective images of the EMS domains

Domain	Image No.	Number of images
Disconnection and rejection	A1, B1	2
Impaired autonomy and performance	A2, B2	2
Impaired limits	A3, B3	2
Other-directedness	A4, B4	2
Overvigilance and inhibition	A5, B5	2

### Confirmatory factor analysis through structural equation modeling

**Fig. 1:**
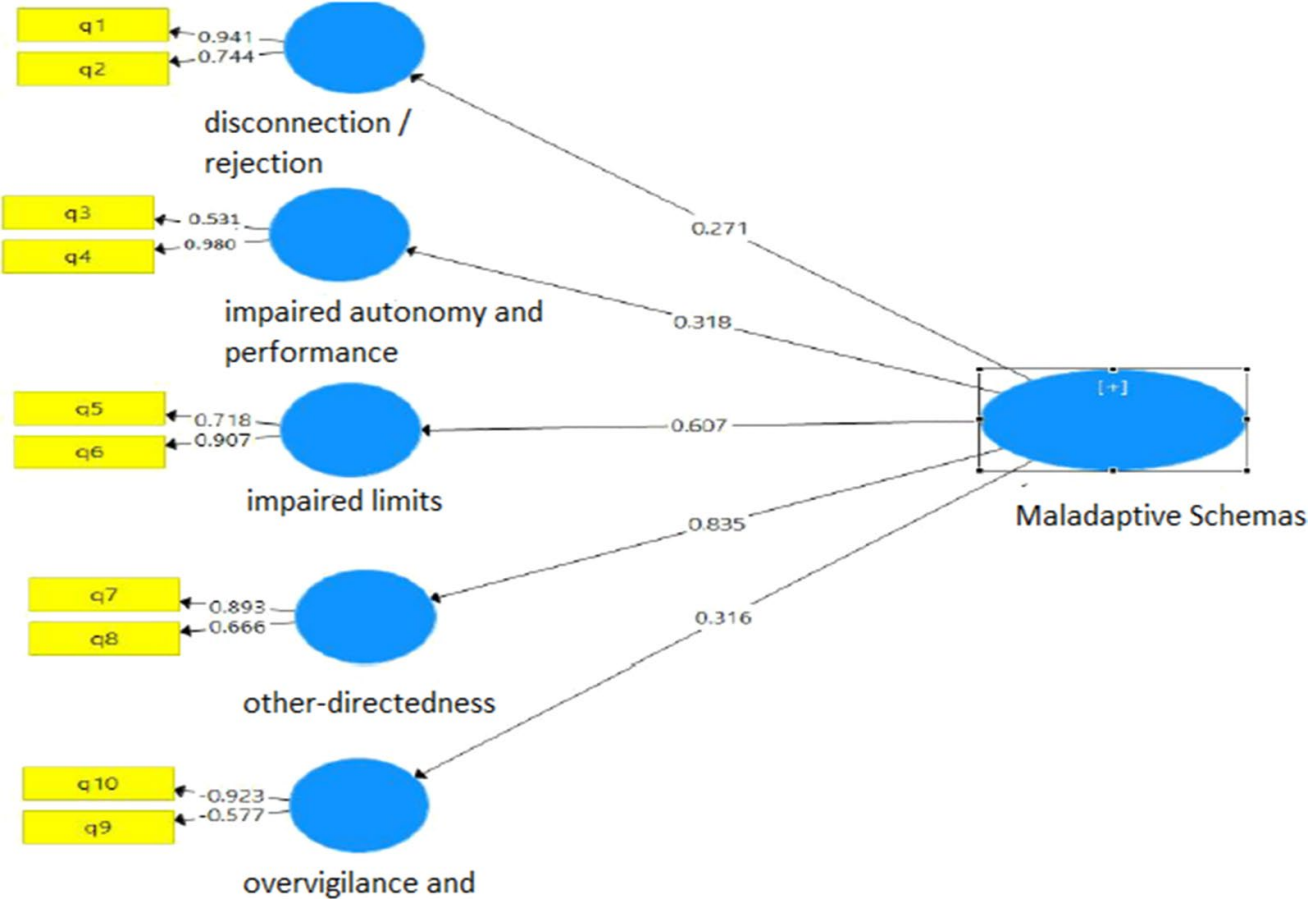
The fitted model of the projective images of Young’s EMS domains in this study

The structural equation modeling technique was used with the partial least squares approach in order to evaluate the proposed model. According to the results of model fitting, the factor loads of all images in the domains were greater than 40%, thereby being significant. This shows the good construct validity of the projective images of the EMS domains Fig. 1.

According to Table 5, the AVE of all variables were above 50%, a finding which indicates that the AVE in the model is acceptable and that the convergent validity in the fitted model is acceptable. Cronbach’s alphas of all variables of the model were higher than 40%. This shows the accuracy of the model. The CR column represents the composite reliability of the model, which indicates that all values are greater than 50%, therefore, the model has an acceptable goodness of fit [19].

**Table 5:** Fitness (Goodness of Fit) Indices for the fitted pattern

Variable	AVE	Cronbach’s alpha	CR
Disconnection and rejection	0.72	0.645	0.835
Impaired autonomy and performance	0.582	0.402	0.507
Impaired limits	0.669	0.528	0.80
Other-directedness	0.62	0.411	0.762
Overvigilance and inhibition	0.593	0.459	0.734

Since the AVE values of the matrix are greater than the correlation values of the variables, it can be stated that the model has appropriate divergent validity and that the measurement model has acceptable goodness of fit (Table 6).

**Table 6:** Fornell–Larcker Matrix to examine the divergent validity of the model

Variable	Disconnection and rejection	Impaired autonomy and performance	Impaired limits	Other-directedness	Over vigilance and inhibition
Disconnection and rejection	0/849				
Impaired autonomy and performance	0/056	0/694			
Impaired limits	0/148	0/327	0/818		
Other-directedness	0/028	0/177	0/187	0/788	
Over vigilance and inhibition	0/024	0/188	0/011	0/127	0/77

### Content and face validities of images

To calculate the content and face validities of the designed images, various projective images were first presented in each domain to the supervisor, who examined the designs in terms of content and projection and made certain points considered in the next images. Finally, ten relatively appropriate images, which were also approved by the supervisor, were then selected. After that, the images were distributed to eleven faculty members and psychologists of Tehran universities along with a survey questionnaire in order to examine their content and face validities. The resultant data of the survey questionnaire were then analyzed through the Lawshe formula in order to obtain the content validity ratio (CVR) [53] reported for each card separately in Table 7. The acceptable content validity for a measurement tool is equal to and greater than 0.63 [4]. The content validity ratio of the designed images was obtained 0.667, a value which indicates an acceptable CVR for a tool.

**Table 7:** The content validity coefficient of each of the designed projective cards

Card	Agreed views	Content validity coefficient
CARD1A	11	1
CARD1B	8	0.45
CARD2A	8	0.45
CARD2B	10	0.81
CARD3A	8	0.45
CARD3B	7	0.27
CARD4A	10	0.81
CARD4B	10	0.81
CARD5A	10	0.81
CARD5B	10	0.81

Question 2—Do the images designed in the domains of disconnection and rejection, impaired autonomy and performance, impaired limits, other-directedness, and over-vigilance and inhibition have sufficient validity?

As discussed earlier, the designed images generally had good construct validity and accounted for 70.35% of the variance of Young’s maladaptive schemas. The images of all five domains had good convergent validity and divergent validity. Considering the research questions, the researcher assessed the convergent and divergent validities of the images designed in each of the five domains based on the correlation between the scores obtained from the images and the scores obtained from the 90-item Young’s EMS-3 for each domain.

The correlation between the scores of the images and the scores obtained from the questions related to the domains were determined to obtain the validity of the images designed in the domains of disconnection and rejection, impaired autonomy and performance, impaired limits, other-directedness, and over-vigilance and inhibition. The results showed that Pearson correlation coefficients between the scores obtained from the images in the domains of disconnection and rejection, impaired autonomy and performance, inverted limits, other-directedness, and over-vigilance and inhibition and the scores of Young’s EMS questionnaire were 0.353, 0.333, 0.346, 0.242, and 0.219, respectively, with the significance level of 0.001. As a result, there is a direct significant correlation between the scores obtained from these two tools. Therefore, the images designed in this study had appropriate convergent validity with Young’s EMS questionnaire items in the introduced domains, and it can be stated that the designed images had sufficient validity Table 8.

**Table 8:** Pearson Correlation Coefficient between the score obtained from the images designed in the domains of disconnection and rejection and the score of Young’s EMS questionnaire items in this domain

Variables	Early maladaptive schemas				
	r	n	p	R^2^	Relationship type
Disconnection and rejection	0.353	102	0.001	0.125	Direct
Impaired autonomy and performance	0.333	102	0.001	0.111	Direct
Impaired limits	0.346	102	0.001	0.12	Direct
Other-directedness	0.242	102	0.001	0.058	Direct
Overvigilance and inhibition	0.219	102	0.001	0.048	Direct

### Descriptive statistics

Table 9 shows the descriptive statistics including the maximum and minimum scores, mean value, standard deviation, and coefficients of kurtosis and skewness for all the ten mentioned cards.

**Table 9:** Descriptive statistics

	Minimum	Maximum	Mean	Std. deviation	Skewness	Kurtosis
Card1A	0	5	2.22	1.332	− .355	− .725
Card1B	0	5	2.52	1.097	− .556	.307
Card2A	0	4	1.73	1.236	.028	− 1.033
Card2B	0	4	1.92	1.200	− .303	− .939
Card3A	0	5	.67	1.093	1.720	2.642
Card3B	0	5	1.44	1.332	.424	− .917
Card4A	0	5	1.25	1.389	.792	− .223
Card4B	0	5	1.38	1.267	.404	− .773
Card5A	0	5	1.61	1.429	.411	− .947
Card5B	0	5	1.73	1.463	.201	− 1.135

## Discussion

The exploratory factor analysis was used on the ten designed cards to extract five factors with the highest variance (70%). The cards of each domain, which had been designed for a specific concept, had the greatest factor load on one of the five factors. In addition, the confirmatory factor analysis for assessment of the proposed five-factor model showed that all of the designed images had a factor of above 40% with their relevant domains or factors. This indicates the construct validity of each of the cards. Another interesting finding was the appropriate discriminant (divergent) validity of each domain with other domains, something which shows different themes and contents of the images of the domains. Therefore, it can be claimed that the research expectations of the cards of each domain were met.

According to the research results, the images designed for the disconnection and rejection domain had a content validity coefficient of 62%, and the exploratory and confirmatory factor analysis indicated the construct validity of the images designed for this domain. Moreover, calculating the convergent validity coefficient between the cards of the disconnection/rejection domain of YSQ-S3 showed a direct and significant correlation coefficient of 0.353. This indicates the good validity of the images designed for this domain. Moreover, these cards can be used effectively to measure it. Simona [55] analyzed the psychometric properties of the YSQ-S3 questionnaire and obtained this domain and its related schemas as the extracted factor with Cronbach's alphas ranging from 80 to 86% as well as good discriminate validity. Lyrakos [32] confirmed this domain and its related schemas among healthy, sick, and recovered individuals with a Cronbach’s alpha ranging between 91 and 98% as well as good validity. The results of these and other studies confirm the construct validity of this domain and its schemas, a finding which is consistent with Young’s theory and the third version of Young’s Schema Questionnaire. The present study also shows the construct and convergent validities of the YSQ-S3 questionnaire. In the conceptual and empirical confirmation of the disconnection and rejection domain based on the results, it can be stated that the impaired limits domain is formed in families which are very nonchalant and overly kind. However, the root of this domain (the deserving schema) is not excessive affection in some cases but a form of extreme compensation for the schemas of the disconnection and rejection domain (emotional deprivation) [67]. Shorey et al. [52] found that the impaired limits and disconnection and rejection domains had a positive correlation with antisocial personality disorder. Wang et al. [65] observed in a 9-year follow-up study on depressed patients that there were correlations of 50% and 60% between the disconnection and rejection domain (mistrust, abuse, and social exclusion schemas) and the impaired limits domain (the deserving schema). Despite the intense control of depression, Tezel et al. [61] showed that maladaptive communication style had a significant positive correlation with the disconnection and rejection and impaired limits domains, however, the controller communication style was not affected by the disconnection and rejection domain. Turner et al. [64] conducted a study on schemas and parental bonding between overweight and normal adolescent girls and found that the subscale of maternal care in the *Parental Bonding Instrument* (*PBI*) had a significant negative correlation with the disconnection and rejection domain (emotional deprivation and mistrust/abuse schemes), the impaired limits domain (the deserving schema), and the over-vigilance and inhibition domain (the emotional inhibition and unrelenting standard schemas). There was also a significant negative correlation between the maternal care level and negative beliefs and the over-vigilance and inhibition domain (the emotional inhibition and unrelenting standard schemas).

The research results showed that the images designed for the impaired autonomy and performance domain had a content validity coefficient of 25%, whereas the exploratory and confirmatory factor analysis confirmed the construct validity of the images designed for this domain. In fact, calculating the convergent validity coefficient between the cards of the impaired autonomy and performance domain of YSQ-S3 indicated a direct and significant correlation coefficient of 0.333. This shows the good validity of the images designed for this domain. In addition, these cards can be used effectively to measure the validity. Saggino et al. [45] analyzed an Italian community and reported that that the YSQ-L3 scale was properly valid, reliable, and capable of discerning the healthy and unhealthy individuals from one another.

Kriston et al. [27] analyzed the validity of the German version of the questionnaire and extracted 18 schemas and five domains from component analysis. They showed that Cronbach's alpha of the impaired autonomy and performance domain of the German version ranged from 40 to 80%. The factor analysis of the model also indicated the domain and schema confirmation. Analyzing the convergent validity of this domain with the SCL-K-9 scale showed a correlation coefficient of 60% and good discriminant validity. This component and domain was also extracted in the present study, indicating good convergent validity, a finding which reflects the internal consistency of the images with Young’s theory as confirmed by other studies of different societies.

In addition, analyzing the evolutionary roots of the impaired autonomy and performance domain shows that parents are over-caring for their children and interfere excessively with their children's personal affairs by undermining their self-esteem and preventing them from forming an independent identity in order to manage their own problems autonomously [67]. Therefore, the maladaptive coping responses of these individuals against the impaired autonomy and performance domain (vulnerability to harm and illness schema) appear in the form of extreme compensation, submission, or avoidance with the schemas of the over-vigilance and inhibition domain (unrelenting standard, negativism-pessimism, emotional inhibition, and punishment schemas). Shorey et al. [52] found that the impaired autonomy and performance and over-vigilance and inhibition domains both correlated with the symptoms of borderline personality disorder. These two domains were also associated with slight signs of antisocial personality disorder in regression analyses. However, people with antisocial disorders had achieved very high scores in the two domains. A review study proved the schema therapy to be efficient in treating borderline personality disorder [25, 49]. A study of 323 participants with personality disorders (including the borderline personality disorder) within the 2006–2011 period in multiple psychological clinics in the Netherlands revealed that the patients treated with schema therapy were less depressed [7]. Renner et al. [42] reported that schema therapy was a good treatment for chronic depression. Moreover, early maladaptive schemas contribute to the vulnerability of chronically-depressed individuals, hence, it is beneficial to focus on them for treating chronic depression [18]. Sundag et al. [58] found that the undeveloped self-schema (impaired autonomy and performance domain) and the defectiveness and shame schema (disconnection and rejection domain) were correlated with the intensified mistrust. The results also showed that the images designed for the impaired limits domain had a content validity coefficient of 0.125, whereas the exploratory and confirmatory factor analyses confirmed the construct validity of the images designed for this domain. Moreover, calculating the convergent validity coefficient between the cards of the impaired limits domain of YSQ-S3 indicated a direct and significant correlation coefficient of 0.346. This indicates the good validity of the images designed for this domain and that these cards can be used efficiently in measuring the validity. In parallel with the results of this study, Di Francisco [15] also extracted 18 schemas in the United States to normalize the *Young’s Schema Questionnaire* and found a significant difference between the healthy group and the patient group in terms of their scores in this schema. This study also confirmed the findings of other studies conducted in other communities with regard to the extraction of these factors and domains. The present study yielded the same results. Moreover, Thimm [63] concluded that the agreeableness personality factor was significantly associated with the impaired limits, disconnection, and rejection and other-directedness domains. D'Onofrio [12] found that the insufficient self-control and self-discipline schema was a predictor of health risk factors. Faustino and Vasco [17] found that the impaired limits domain acted as a mediator for the correlation of emotional processing difficulties and psychological needs. This can explain the findings of Malogiannis et al. [33], who reported that 60% of the chronically-depressed individuals responded satisfactorily to schema therapy. They also indicated that the treatment’s effects lasted in a follow-up study six months after the completion of the treatment.

The results indicated that the images designed for the other-directedness domain had a content validity coefficient of 62% and that the exploratory and confirmatory factor analyses confirmed the construct validity of the images designed for this domain. In addition, calculating the convergent validity coefficient between the cards of the other-directedness domain of YSQ-S3 showed the direct and significant correlation coefficient of 0.242. This indicates the good validity of the images designed for this domain, whereas these cards can be used efficiently to measure the validity. Similar results were obtained in a study in Korea, which extracted five domains and 18 schemas, defined by Young, with greater internal correlation (97%) than other studies conducted on the Turkish versions (63–80%), Arabic versions (70–88%), and the second version of Young’s questionnaire in Korea for 13 schemas (72–90%). The third Korean version of YSQ-S3 with SCL-90, BDL, and ECR-M scales was also significantly correlated, and this domain also had good validity and reliability. The projective cards of the present study also showed good construct, criterion and content validity. Calvete et al. [9] showed that the other-directedness domain was related to the change and endurance of social anxiety. Balsamo et al. [6] revealed that the other-directedness domain mediated between co-rumination and depression. Alba et al. [1] reported that the other-directedness domain mediated between being a victim of bullying and the trajectory of symptoms. This schema domain also predicted anger and internal aggression as well as internalized symptoms such as anxiety and phobia [20].

According to the results, the images designed for the over-vigilance and inhibition domain had a content validity coefficient of 75%. Moreover, the exploratory and confirmatory factor analyses indicated the construct validity of the images designed for this domain. Calculating the convergent validity coefficient between the cards of the over-vigilance and inhibition domain of YSQ-S3 showed the direct and significant correlation coefficient of 0.219. This shows the good validity of the images designed for this domain. Furthermore, these cards can be used efficiently in measuring the validity. [22] conducted a study in Tehran to analyze the psychometric properties of the YSQ-S3 scale between a healthy group and a patient one. They obtained five basic factors and 15 schemas and reported that there was a significant correlation between this scale and inefficient attitudes. Renner et al. [41] proposed a model of underlying risk factors for chronic depression (early trauma, cognitive factors, personality pathology, and interpersonal factors). They employed schema therapy to treat the abovementioned risk factors of chronic depression. The results indicated that ST was successful in the treatment of chronic depression. Curley et al. [11] found that individuals with intense trauma in childhood benefited the most from schema therapy. In a review study, Nicol et al. [35] observed a correlation between early maladaptive schemas and psychological pathology in the youth, something which can distinguish between depression, anxiety, eating disorder, symptomology of personality disorders, and external behavior. Sunde et al. [59] conducted a study on OCD patients and reported that the effects of the exposure response prevention (ERP) treatment approach would be improved by targeting the maladaptive schemas of this group for treatment. They also reported that those with numerous maladaptive schemas, who normally did not respond to ERP, would also benefit from it. Kunst et al. [29] found that the over-vigilance and inhibition domain correlated with the obsessive–compulsive disorder. In addition, dos Santos Ribas et al. [16] found a significant correlation between over-vigilance and inhibition domains (schemas of unrelenting standards, and hyper criticalness) in migraine patients from both genders. Grigorian et al. [23] found that this domain was negatively correlated with drug abuse awareness and control.

According to the review studies, it is possible to confirm the schema domains construct in both empirical and conceptual contexts. The findings of this study and the validation of the YSQ-S3 scale are consistent with the findings of other studies on Young’s schema domains as well as early maladaptive schemas.

## Conclusions

The results indicated that the designed projective images yielded acceptable construct validity. It is suggested that a similar study with a greater sample size be conducted on other clinical samples in order to examine further evidence by comparing the validity of the images with other psychological scales.

## Supplementary Information

**Additional file 1.** Images data.

## Data Availability

Data are attached as Additional file 1, and the information related to the study is available in the manuscript. The images of this study were also added to an “Appendix” for further use in other studies.

## Appendix

This study seeks to design and validate ten projective images related to Young’s EMS domains, designing two cards A and B for each domain. “A” cards are based on individuals’ basic emotional needs and early life experiences, and “B” cards are also based on their basic emotional needs and everyday life situations in adulthood.

### Images and Cards 1 (Disconnection and rejection domain)

The subject of 1A card is about emotional need of attachment.

The subject of 1B card is about a projection of the everyday life situation that related to the concept of emotional need of attachment.

### Images and Cards 2 (Impaired autonomy and performance domain)

The subject of 2A card is about emotional need of autonomy and competence.

The subject of 2B card is about a projection of the everyday life situation that related to the concept of emotional need of autonomy and competence.

### Images and Cards 3 (Impaired limits domain)

The subject of 3A card is about emotional need of realistic limits and self-control.

The subject of 3B card is about a projection of the everyday life situation that related to the concept of emotional need of realistic limits and self-control.

### Images and Cards 4 (Other-directedness domain)

The subject of 4A card is about emotional need of freedom to express valid needs and emotions.

The subject of 4B card is about a projection of the everyday life situation that related to the concept of emotional need of freedom to express valid needs and emotions.

### Images and Cards 5 (Over vigilance and inhibition domain)

The subject of 5A card is about emotional need of spontaneity and play.

The subject of 5B card is about a projection of the everyday life situation that related to the concept of emotional need of spontaneity and play.

## Abbreviations

EMS: Early maladaptive schema
TAT: Thematic apperception test
CAT: Children's apperception test
SAT: Senior appreciation test
TPPC: Pictorial test of cognitive profiles
PPM: Photo projective method
SQ-SF: Questionnaire-short form
PAF: Principal axis factoring
KMO: Kaiser–Meyer–Olkin test
AVE: Average variance extracted index
EFA: Exploratory factor analysis
YSQ-S3: Young Schema Questionnaire Short Form 3
YSQ-L3: Young Schema Questionnaire Long Form 3
SCL-K-9: Symptom-Checklist-K-9
BDL: Backstrand Dahlberg Liljenas Balance Scale
OCD: Obsessive compulsive disorder
ERP: Exposure response prevention
CVR: Content validity ratio
